# A comparative analysis of *Wolbachia*‐induced host reproductive phenotypes reveals transition rate heterogeneity

**DOI:** 10.1002/ece3.3789

**Published:** 2018-01-15

**Authors:** Heinrich zu Dohna, Carine Houry, Zakaria Kambris

**Affiliations:** ^1^ Department of Biology American University of Beirut Beirut Lebanon

**Keywords:** comparative methods, host reproductive phenotype, phylogenetics, *Wolbachia*

## Abstract

The endosymbiotic bacterium *Wolbachia* infects a wide range of arthropods and their relatives. It is an intracellular parasite transmitted through the egg from mother to offspring. *Wolbachia* can spread and persist through various means of host reproductive manipulation. How these different mechanisms of host manipulation evolved in *Wolbachia* is unclear. Which host reproductive phenotype is most likely to be ancestral and whether evolutionary transitions between some host phenotypes are more common than others remain unanswered questions. Recent studies have revealed multiple cases where the same *Wolbachia* strain can induce different reproductive phenotypes in different hosts, raising the question to what degree the induced host phenotype should be regarded as a trait of *Wolbachia*. In this study, we constructed a phylogenetic tree of *Wolbachia* and analyzed the patterns of host phenotypes along that tree. We were able to detect a phylogenetic signal of host phenotypes on the *Wolbachia* tree, indicating that the induced host phenotype can be regarded as a *Wolbachia* trait. However, we found no clear support for the previously stated hypothesis that cytoplasmic incompatibility is ancestral to *Wolbachia* in arthropods. Our analysis provides evidence for heterogeneous transition rates between host phenotypes.

## 1 INTRODUCTION

*Wolbachia* is one of the most common intracellular parasitic bacteria found in arthropod species and their relatives. Estimates of the percentage of insect species infected by *Wolbachia* range from 40% to 65% (Hilgenboecker, Hammerstein, Schlattmann, Telschow, & Werren, 2008; Zug & Hammerstein, 2012). There is a considerable interest in *Wolbachia* due to its wide host range, its complex interaction with its hosts, and its potential as biological control agent (Augustinos et al., 2011; Iturbe‐Ormaetxe, Walker, & O’ Neill, 2011; Kageyama, Narita, & Watanabe, 2012; Slatko, Taylor, & Foster, 2010).

*Wolbachia* is maternally transmitted to offspring through the egg's cytoplasm. Several *Wolbachia* strains increase their spread in arthropod populations by manipulating their host's reproductive phenotype, often at the expense of the host's fitness (Werren Baldo, & Clark, 2008). These manipulations include, among others, the selective killing of male offspring (MK), the induction of parthenogenesis (PI), the feminization of genetic males (FI), and the induction of cytoplasmic incompatibility (CI) which reduces the offspring viability in a cross between an uninfected female and an infected male (Werren et al., 2008). These different manipulations of the host's reproduction will be referred to as the *host reproductive phenotype* in the subsequent text.

How *Wolbachia* manipulates the host on a molecular level to induce a particular host reproductive phenotype and how *Wolbachia* evolved such a wide array of different host reproductive phenotypes are largely unknown (Werren et al., 2008). A recent study has shown that *Wolbachia* supergroups A and B, the two supergroups that contain the largest diversity of hosts and host reproductive phenotypes, form a monophyletic clade (Gerth, Gansauge, Weigert, & Bleidorn, 2014). Consequently, the ability to adapt to a wide range of hosts and to trigger many different host reproductive phenotypes appears to have a single evolutionary origin (Gerth et al., 2014). The host reproductive phenotype associated with this origin, that is the host reproductive phenotype ancestral to supergroups A and B, is unknown. It has been suggested that CI is ancestral to all Wolbachia lineages (Rousset, Bouchon, Pintureau, Juchault, & Solignac, 1992; Stouthamer, Breeuwer, & Hurst, 1999). However, to the best of our knowledge, this assertion has never been analyzed using comparative methods.

The identification of the host reproductive phenotype ancestral to supergroups A and B might provide a clue to the evolutionary mechanism that led to the successful expansion of *Wolbachia*. Furthermore, the evolutionary transition rates between different host reproductive phenotypes can provide clues about the molecular mechanisms producing these host phenotypes, as a high rate of evolutionary transitions between two host reproductive phenotypes could indicate that these phenotypes are triggered by shared underlying mechanisms. There are at least two unrelated examples where one *Wolbachia* strain induces CI and its closely related sister strain induces MK (Hornett et al., 2008; Jiggins, Bentley, Majerus, & Hurst, 2002), two additional examples where the induced host phenotype changes from CI to MK when a *Wolbachia* strain is transfected into a different host (Jaenike, 2007; Sasaki & Ishikawa, 2000), and an example of a PI‐inducing *Wolbachia* strain that shows evidence for vestigial CI (Kraaijeveld & Reumer, 2011). However, it is unclear whether these examples are indicative of a general high transition rate between CI and MK, or between CI and PI across the phylogenetic tree of *Wolbachia*. The transfection experiments indicate that sometimes the change between different host reproductive phenotypes is not due to genetic changes in *Wolbachia*, and they raise the question whether the host reproductive phenotype should even be considered a *Wolbachia* trait.

The purpose of this study was to address three interrelated questions about the evolution of host reproductive phenotype in *Wolbachia*, namely whether the host reproductive phenotype can be considered a trait of *Wolbachia*, what is the most likely ancestral host reproductive phenotype of supergroups A and B, and whether the transition rates between some host reproductive phenotypes are higher than others. We used comparative methods to address these questions. We constructed phylogenetic trees of *Wolbachia*, analyzed the phylogenetic signal of host reproductive phenotype on these trees, estimated the ancestral host reproductive phenotype, and estimated transition rates between host reproductive phenotypes along that tree.

## 2 MATERIALS AND METHODS

### 2.1 Sequence collection and alignment

Sequences for the six loci *gatB*,* coxA*,* hcpA*,* fbpA*,* ftsZ*, and *wsp* were downloaded from the MLST *Wolbachia* database (http://pubmlst.org/wolbachia/ on 28 April 2016). This database stores, in addition to sequence data, information about the host species, sampling location, infection status, host reproductive phenotype, and more (Baldo et al., 2006). Only sequences of strains with a determined host reproductive phenotype from hosts with single *Wolbachia* infection were selected. Host reproductive phenotype entries in the database are either *CI* (cytoplasmic incompatibility), *MK* (male killing), *FI* (feminization), *PI* (parthenogenesis), “other,” or blank. At least one submitter used host reproductive phenotype “other” for an unknown phenotype (M. Ramalho, personal communication). The database imposed a trade‐off between the stringency of the inclusion criteria and the number of taxa included in the study. To avoid this trade‐off, we performed the complete set of analyses on two sets of taxa, a smaller set of taxa with more stringent inclusion criteria and a larger set with less stringent criteria. The more stringent taxon set included all strains that had the loci *gatB*,* coxA*,* hcpA*,* fbpA*,* ftsZ,* and *wsp* sequenced. Strains with host reproductive phenotype “other” were only included in this set if we could find publications of *Wolbachia* infections that matched the host species and location of the database entries and analyzed the host reproductive phenotype. In one such case, the host reproductive phenotype was increased female fecundity (Zhang, Zhang, & Hong, 2010), and in two cases, the host was tested for all known reproductive phenotypes but showed none (Hamm et al., 2014). In all of these cases, the entries were retained and phenotype “other” was used. Entries with host reproductive phenotype “other” with no description in the literature were removed. This led to the inclusion of 53 strains in the more stringent set. The less stringent set included all strains that had the loci *gatB*,* coxA*,* hcpA*,* fbpA*, and *ftsZ* sequenced and all strains with host phenotype “other,” leading to the inclusion of 71 strains. An isolate of the supergroup D with all six loci sequenced was added as an outgroup to both taxon sets. Seven multiple sequence alignments were created per taxon set, one per locus and one for sequences obtained from concatenating all loci. The alignments were estimated using MAFFT version 7 (http://mafft.cbrc.jp/alignment/server/; Katoh & Standley, 2013).

### 2.2 Tree reconstruction

Phylogenetic trees were reconstructed from alignments using MrBayes v.3.2 (Ronquist et al., 2012), selecting a general time reversible substitution model with a proportion of invariable sites and a gamma‐shaped distribution of substitution rates across sites (GTR + I + Γ). Two Monte Carlo Markov chains were run to generate samples from posterior distributions of trees and substitution rate parameters, given the alignments. The chains were run for one million generations, and if the standard deviation of split frequencies did not fall to 1% or below, the chains were run for 10 million generations. In both cases, a sample frequency of 500 was used. Trees were estimated for the alignment of single locus and for the alignment of concatenated sequences.

### 2.3 Maximum likelihood rate estimation

The phylogenetic trees were used to estimate transition rates of *Wolbachia* strains between different host phenotypes. A multistate model was fitted where the host phenotype was treated as a trait of *Wolbachia* with five different states, CI, MK, FI, PI, and O for “other.” Transition rates between different host phenotypes in the multistate model were fitted via maximum likelihood to the consensus trees obtained from the MrBayes runs. Eight different models were fitted per consensus tree. The first three models were a model with a single rate for all transitions, a symmetric model where for all pairs of phenotypes *i* and *j,* the transition rates from phenotype *i* to *j* were constrained to be equal to the rates from *j* to *i*, and an unconstrained model with a free parameter for each of the 20 different transition rates. In addition, five models with 1, 2, 3, 4, or 5 free‐rate parameters were fitted. These models took the unconstrained model fit as starting point and set all rates to zero that were <0.1 according to the unconstrained model fit. All other rates were grouped into 1, 2, 3, 4, or 5 rate classes by dividing the entire range of estimated rates into evenly spaced intervals and assigning all rates in the same interval to the same rate class. Of these eight models, the one with the lowest Akaike information criterion (AIC) was chosen as the best‐fitting model. This analysis was performed for the consensus tree based on the concatenated sequences and the locus‐specific consensus trees, using the R package *geiger* (Harmon, Weir, Brock, Glor, & Challenger, 2008).

### 2.4 Estimating a phylogenetic signal for host phenotypes

Two methods were used to estimate the phylogenetic signal of host phenotypes. According to one method, transition rates between different host phenotypes in the multistate model were fitted via maximum likelihood to a star tree (i.e., a tree with no phylogenetic structure), using the R package *geiger* (Harmon et al., 2008). The difference in AIC between the model fitted to the star tree and the model fitted to the actual tree provides an estimate for the strength of the phylogenetic signal of host phenotypes. In addition, the minimum number of transitions between the different host phenotypes was estimated based on the consensus tree. This minimum number of transitions was compared with a null distribution that was obtained by randomly permutating the host phenotypes on the consensus tree and estimating the minimum number of transitions for each permutation. The permutation analysis was performed using the R function *phylo.signal.disc* that was developed for a previous analysis (Bush et al., 2016). Both analyses, the comparison with the star tree and the permutation analysis, were performed for the alignment of each locus and the alignment of the concatenated sequences.

### 2.5 Rate estimation via a reversible jump MCMC

In addition to the maximum likelihood method described above, we employed Bayesian Monte Carlo Markov Chains (MCMC) to estimate transition rates of *Wolbachia* strains between different host reproductive phenotypes. The phylogenetic trees based on the concatenated sequences were used as input for this estimation procedure. Posterior distributions of the transition rates of strains between the different host phenotypes were estimated using the software package *BayesTraits* (Pagel, Meade, & Barker, 2004). Five different reversible jump MCMCs were run with an exponential prior distribution for all rate parameters with means 1, 10, 100, and 500 for the smaller taxon set and with a mean of 100 for the larger taxon set. The number of generations was 500 million for all five runs. The reversible jump procedure produces samples from the posterior distributions of parameters and models. When the BayesTraits MCMC explores the model space, it sets some rates to zero, sets the number of free parameters (i.e., number of rate classes) for the remaining rates, and distributes them over the rate classes (Pagel & Meade, 2006). For example, a model in which some transition rates are zero and all nonzero rates are equal to each other is a model with one free parameter, where all nonzero rates belong to the same rate class. We classified the models returned by the MCMC by their number of free parameters and calculated a Bayes factor for each model and for each model class (for details, see Appendix S1). To obtain an overall ranking of transition rates across different estimation methods (Bayesian vs. maximum likelihood and small vs. large taxon set), we standardized the rate estimates to be between zero and one for each taxon set and method combination and then averaged for each transition the standardized rate estimates across methods and taxon sets.

Convergence of parameter distributions to a stationary distribution was tested using Geweke's convergence diagnostic (Geweke, 2005; R package *coda*). Ancestral state probabilities were estimated for the supergroups A and B and the clade that contains supergroups A and B, using the *BayesTraits* command “AddMRCA.” Calculation of convergence diagnostics, variance analysis, and tree plotting were carried out in R (R Core Team, 2015).

## 3 RESULTS

**Figure 1:**
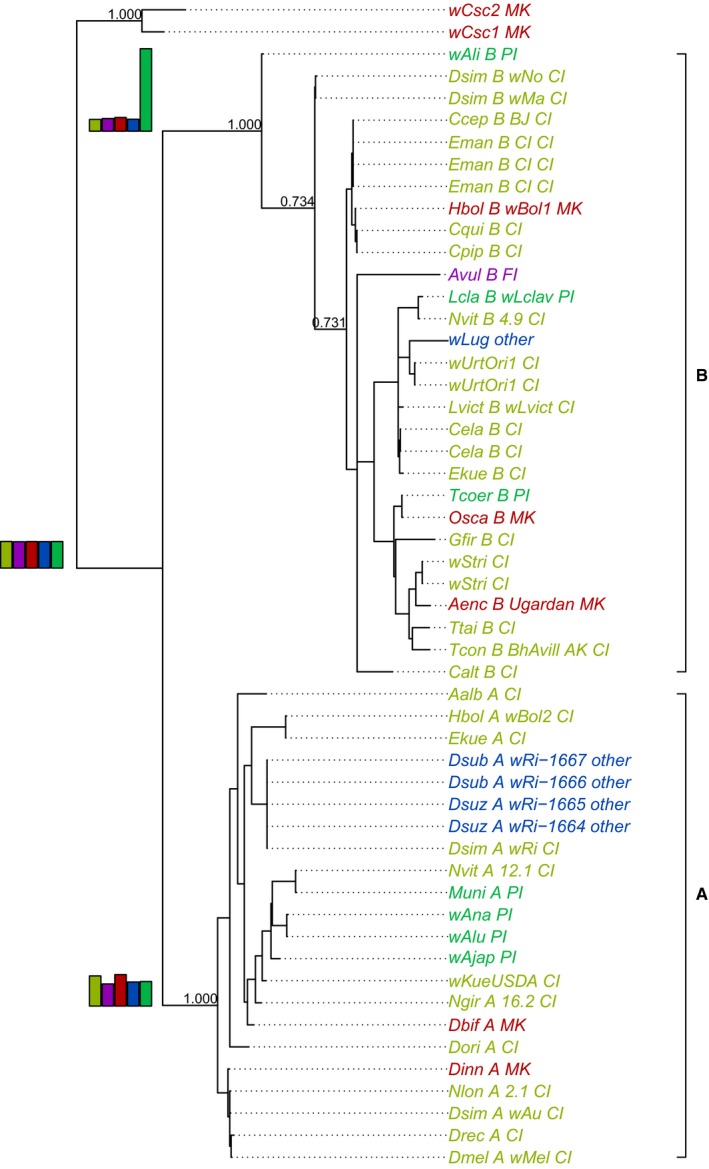
Phylogeny estimated by Bayesian MCMC from an alignment of the six concatenated genes *gatB*,* coxA*,* hcpA*,* fbpA*,* ftsZ,* and *wsp*. Tip labels indicate strain and host phenotype (CI = cytoplasmic incompatibility, MK = male killing, FI = feminization induction, PI = parthenogenesis induction). Tip labels are colored according to host phenotype. Posterior probability values are shown at major nodes and brackets on the right show supergroups A and B. Bar graphs at the nodes for the two supergroups and the root show the posterior probabilities for the ancestral host phenotype. Color coding for bar graph is the same as tip labels

The results produced by both sets of analyses were largely consistent. Unless stated otherwise, the following text reports the results of the analysis with six loci and 53 strains. The phylogenetic tree based on the concatenated genes resolved the *Wolbachia* supergroups A and B with posterior probabilities of one (Figure 1). There were frequent transitions between the different phenotypes along the tree. All host reproductive phenotypes were about equally likely to be ancestral for the clade that combines supergroups and A and B. The most likely ancestral host phenotypes for supergroups A and B depended on the taxon set analyzed. The most likely ancestral phenotypes for supergroups A and B were MK and PI, respectively, in the smaller taxon set (Figure 1), and CI for both supergroups in the larger taxon set (results not shown). According to the maximum likelihood estimation, the model with four free‐rate parameters fitted to the tree based on concatenated loci achieved the best fit (i.e., lowest AIC, Table 1). The maximum likelihood models fitted to trees based on individual loci generally produced a worse fit (higher AIC) than the model fitted to a tree based on the concatenated loci, and the fit was worst (AIC was highest) for a transition model fitted to a star tree, that is, to a tree without phylogenetic structure (Table 1). The minimum number of transitions between host reproductive phenotypes was lowest along the *Wolbachia* tree based on concatenated loci (Table 1) and was significantly lower than when host reproductive phenotypes were randomly permutated on the tree (*p* = .0003, Table 1).

**Table 1:** Phylogenetic signals for each locus and for concatenated genes

Tree based on	Number of free parameters of best model	AIC	Minimum number of transitions		*p*‐Value for transitions
			Actual distribution	Permutations (median)	
*wsp*	4	118.2	18	20	.070
*hcpA*	4	118.0	17	20	.015
*ftsZ*	4	115.1	20	20	.64
*fbpA*	3	117.3	18	20	.074
*coxA*	1	114.6	18	20	.073
*gatB*	4	127.2	17	20	.016
Concatenated genes	4	111.2	15	20	.0003
Star tree	–	132.1	–		

**Figure 2:**
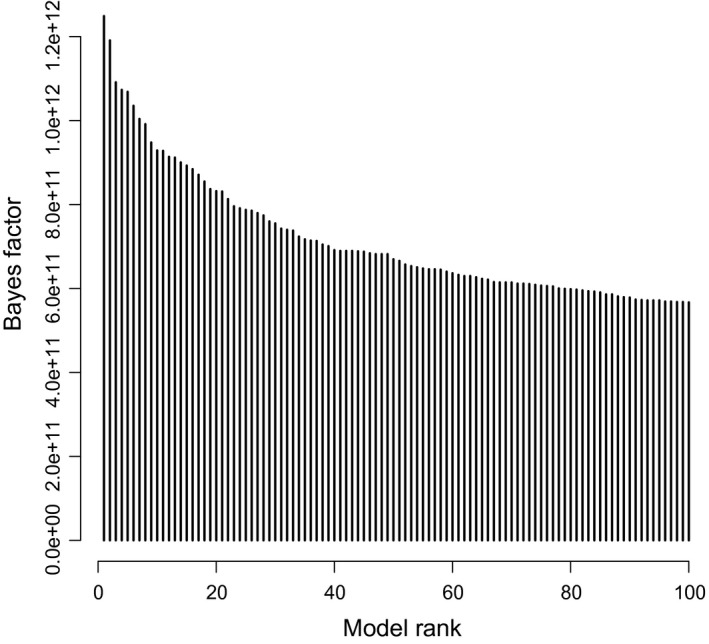
Bayes factors of 100 best models ordered by posterior frequency

The results produced by five different Bayesian reversible‐jump MCMC were similar to each other. As the run with exponential prior mean of 100 (run 3) yielded rate estimates most consistent with the maximum likelihood estimates, any specific numeric results presented in this section will be from this run. All runs produced the highest Bayes factor for the class of models with only one free parameter. Within this class, no model dominated strongly, but instead, posterior odds were spread relatively evenly among a large number of models (Figure 2). In run 3, this class of models had a log(Bayes factor) of 23.0 (Table 2), a value that can be considered “decisive evidence” in favor of this model class (Kass & Raftery, 1995). For all models with more than two free parameters, the Bayes factors were below one. Hence, in contrast to the maximum likelihood analysis, the Bayes factors suggest that models with only one free parameter fit the data best.

**Table 2:** log (Bayes Factor) for models with different number of free parameters

Number of free parameters	log (Bayes Factor)
1	23.0
2	9.4
>2	<0

**Figure 3:**
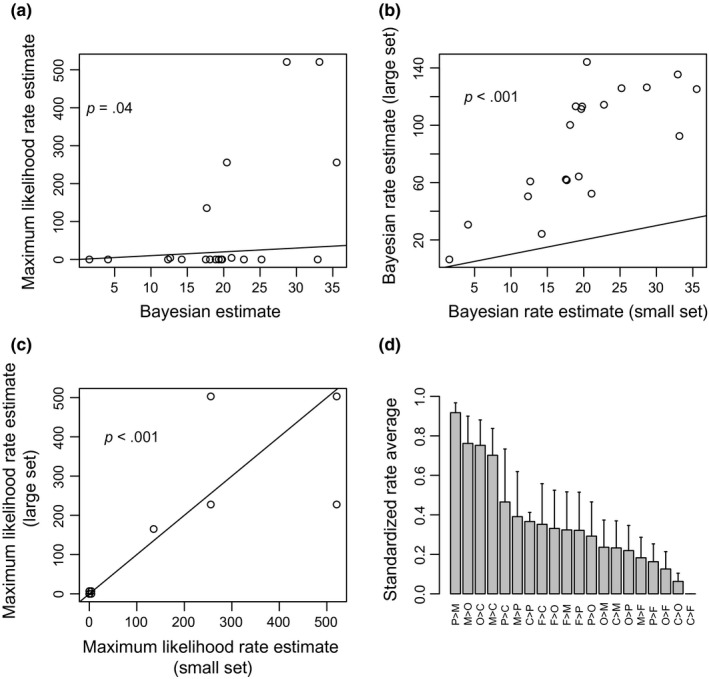
Transition rate estimates according to different methods and datasets. (a) comparison of maximum likelihood and Bayesian rate estimates according to small taxon set, (b) comparison between Bayesian rate estimates in different taxon sets, and (c) comparison between maximum likelihood rate estimates in different taxon sets. Solid lines indicate the 1:1 line. *p*‐Values for test that Spearman correlation differs from zero. (d) Average and standard error of standardized transition rates over all four combinations of dataset and estimation method

Despite the discordance between the Bayesian and maximum likelihood analysis in terms of model complexity, the Bayesian posterior means for transition rates between the host reproductive phenotypes were significantly positively correlated with the maximum likelihood rate estimates (*r *=* *.46, *p *=* *.04, Figure 3a, Table 3). Nevertheless, there was considerable variation between these two estimation methods, and the maximum likelihood method generally produced higher rate estimates (Figure 3a). Convergence tests showed that posterior distributions of all but two rates converged to a stationary distribution. The estimates for transition rates between host phenotypes were correlated between the two taxon sets (six loci for 53 taxa versus five loci for 71 taxa). The correlation between rate estimates was higher when comparing results between datasets within analysis method (maximum likelihood or Bayesian) than between analysis methods within datasets (Table 3). The four highest transition rates when averaging standardized rate estimates over different approaches were PI>MK, MK>O, O>CI, and MK>CI (Figure 3d).

**Table 3:** Correlations between different transition rate estimates

Dataset	Analysis method	Six loci and 53 strains		Five loci and 71 strains	
		Maximum likelihood	Bayesian	Maximum likelihood	Bayesian
Six loci and 53 strains	Maximum likelihood	1	0.46*	0.83**	0.24
	Bayesian		1	0.45*	0.78**
Five loci and 71 strains	Maximum likelihood			1	0.29
	Bayesian				1

*p < .05; **p < .001.

## 4 DISCUSSION

This study investigated the evolution of *Wolbachia's* propensity to induce different reproductive phenotypes in arthropod hosts. The phylogenetic signal of host reproductive phenotypes was weak or absent on locus‐specific *Wolbachia* trees, but it was highly significant when the *Wolbachia* tree was based on concatenated sequences of multiple loci. All host reproductive phenotypes were equally likely to be ancestral to the clade that contains supergroups A and B. When supergroups A and B were analyzed separately, the most likely ancestral host phenotypes for supergroups A and B depended on the taxon set analyzed. The most likely ancestral host phenotypes were MK for supergroup A and PI for supergroup B in the smaller taxon set, and when the larger taxon set was analyzed, CI was the most likely ancestral phenotype for both supergroups. According to maximum likelihood estimates, the best model for the transition rates between host reproductive phenotypes was a model with four free‐rate parameters fitted to the tree based on concatenated loci. According to Bayesian estimates, models with only one free‐rate parameters had the highest support. Bayesian estimates of transition rate between host reproductive phenotypes were positively correlated with maximum likelihood estimates but generally lower.

This study demonstrated a clear phylogenetic signal of host reproductive phenotype on a *Wolbachia* tree based on concatenated sequences, thereby providing a formal justification to treat the host reproductive phenotype as a *Wolbachia* trait. The lack of phylogenetic signal of host reproductive phenotype on most loci‐specific trees suggests that these loci are not linked to genes that are involved in the host phenotype manipulation. The fact that combining these loci produces a clearer phylogenetic signal of host reproductive phenotype shows that host reproductive phenotypes tend to be similar among closely related *Wolbachia* strains. If one assumes that host reproductive phenotypes should show a phylogenetic signal as they are at least partly influenced by *Wolbachia* genetics, then the stronger phylogenetic signal on trees based on concatenated sequences could be interpreted as evidence that concatenating loci provides more reliable estimates of relatedness between strains than individual loci. This is in line with previous studies showing that concatenating loci can provide reliable trees in bacteria (Lang, Darling, & Eisen, 2013).

The models used in this study to estimate transition rates assumed that each *Wolbachia* strain could induce only one reproductive host phenotype. There is evidence from previous studies that a single *Wolbachia* strain can induce more than one host reproductive phenotype (Jaenike, 2007; Sasaki & Ishikawa, 2000). However, our analysis demonstrated a clear phylogenetic signal of host reproductive phenotype. Hence, the ability to trigger multiple host reproductive phenotypes is either not widespread enough to mask the phylogenetic signal or external factors, such as host species, that influence the host reproductive phenotypes are themselves linked to *Wolbachia* genetics. In either case, a model that assumes a single host reproductive phenotype per *Wolbachia* is an approximation that, while ignoring some of the biological complexity, should still capture the dominant patterns.

The models fitted in this study furthermore assume that the rate at which strains switch between a particular pair of phenotypes is constant along the tree. While we did not formally test this assumption, a visual inspection of the *Wolbachia* tree does not suggest a rate heterogeneity.

The most commonly described phenotype induced by *Wolbachia* in arthropods is CI (Atyame et al., 2014), and the same is true for the dataset analyzed here. As the ability to induce CI is found in different *Wolbachia* lineages, it has been suggested that this ability is ancestral (Rousset et al., 1992). Our analysis does not provide clear support for this hypothesis. In both taxon sets, all host reproductive phenotypes appear equally likely to be ancestral to the clade that contains supergroups A and B, and the clade that gave rise to the broad diversity of hosts and host manipulations in *Wolbachia*. The most likely ancestral host reproductive phenotype for each supergroup, A and B, depended on the taxon set and loci included in the analysis. In the smaller taxon set, the ancestral phenotypes of supergroups A and B appear to be strongly driven by individual strains. For example, PI was the most likely ancestral host reproductive phenotype in supergroup B. This result seems to be mainly due to a single PI‐inducing strain that is found in *Diaphorencyrtus aligarhensis* and has a more ancestral sequence among the concatenated loci (Figure 1). This strain did not appear more ancestral in the larger taxon set that did not include the *wsp* locus. In this taxon set, CI was the most likely ancestral phenotype for supergroup B (results not shown). Hence, the ancestral host reproductive phenotypes of the supergroups A and B were sensitive to the strains and loci included in the analysis. The data were therefore not sufficient to confirm the previously stated hypothesis that CI is the ancestral host reproductive phenotype.

The biggest challenge in our analysis was the estimation of transition rates between host reproductive phenotypes. The transition between five phenotypes required the estimation of 20 rate parameters. This large number of parameters had to be estimated based on a modest set of taxa and a noisy signal. To overcome this challenge, we explored different steps to reduce the number of free parameters, analyzed different taxon sets, and employed maximum likelihood estimation and Bayesian reversible‐jump MCMCs, two approaches that have different strengths and limitations. A comparison between the results produced by the different data and methods shows which aspects of the results are robust and which are not. An obvious difference between the maximum likelihood and the Bayesian approaches was that the number of free parameters of the best model was four according to the maximum likelihood procedure and one according to the Bayesian procedure. This discrepancy could be due to the fact that the AIC, the criterion used to compare models in the maximum likelihood framework, compares models at point estimates of parameters that provide the best fit, whereas Bayes factors compare marginal likelihoods of models integrated over the entire range of the prior distribution. Hence, single‐parameter models might be more robust to parameter deviations from the optimum. Alternatively, the discrepancy might have been caused by the fact that the maximum likelihood estimation was based on the consensus tree, whereas the Bayesian method accounted for phylogenetic uncertainty. Whatever its cause, this discrepancy between the models prevents us from drawing any firm conclusions about the optimal number of free parameters. The rate estimates were more sensitive to the estimation method than to the data used, suggesting that there are inherent differences in the methods that consistently lead to different rate estimates, regardless of the idiosyncrasies of the data.

The Bayesian analysis strongly favored models with one nonzero rate parameter, that is, models in which all nonzero rates were equal to each other. No single model dominated strongly within that model class; hence, different models with different rates set to zero had similar support. Different rates occurred among these models in the nonzero rate class at different frequencies such that the posterior means differed between the rates when averaged across these models, even though within each model all nonzero rates are the same.

Averaging across different models in the Bayesian analysis produced posterior means of rate estimates that were closer to the maximum likelihood estimates, even though each Bayesian model with high support had only one free parameter. Hence, the optimal model complexity (i.e., the best number of free parameters) was not consistent between the two approaches, but the estimated rate parameters were positively correlated between the approaches. The four highest transition rates when averaging rate estimates over different approaches were PI>MK, MK>O, O>CI, and MK>CI. Previous studies produced experimental evidence that some *Wolbachia* strains transition easily between inducing MK and CI (Hornett et al., 2008; Jiggins et al., 2002), including cases, where *Wolbachia* strains switch between CI and MK when transfected in different hosts (Jaenike, 2007; Sasaki & Ishikawa, 2000). Even though these examples were not part of the dataset analyzede in this study, the transition rate from MK to CI was consistently identified as high in our analysis. Hence, our study provides additional independent evidence for a high transition rate between MK and CI, indicating that the induction of MK and CI might be based on some shared underlying mechanisms.

Another example for a transition with a consistently high rate according to the two estimation methods is PI>MK. It is less clear how this result relates to previous experimental evidence. A study on the effect of *Wolbachia* in the parasitoid wasp *Asobara japonica* showed that PI involves a feminization step (Ma et al., 2015), suggesting that there should be a high transition rate between PI and FI. However, it has also been shown that MK can occur through lethal feminization (Kageyama & Traut, 2004); hence, it is conceivable that the feminization step that was discovered in PI is functionally related to MK.

In conclusion, the phylogenetic signal of host reproductive phenotype on a *Wolbachia* tree provides a justification for fitting a simple comparative model for *Wolbachia's* evolutionary transitions between different host reproductive phenotypes, despite previous evidence that some complexities of the evolution of host reproductive phenotype manipulation are not captured by such a model. The model fitted here produced some results that were robust with respect to analysis methods and confirmed previous experimental evidence about the ease of transition between MK and CI. We expect that the approach presented here will further contribute to our understanding of *Wolbachia* evolution, once increased data availability will allow the inclusion of more strains and more loci.

## CONFLICT OF INTEREST

None declared.

## AUTHOR CONTRIBUTIONS

HzD and ZK conceived the study. CH and HzD performed the analysis. All authors wrote the manuscript.
