# Supplementary material for: A comparative analysis of Wolbachia‐induced host reproductive phenotypes reveals transition rate heterogeneity
Source: Ecol Evol. 2018 Jan 15;8(4):1945–53. doi: 10.1002/ece3.3789 (PMC5817148; doi:10.1002/ece3.3789)
Supplement: Supplementary file 1 [file ECE3-8-1945-s001.docx]

**Appendix S1**

*Bayes factor calculations*

The reversible-jump mode of BayesTraits performs transitions between different parameter values and between different models, i.e. different sets of free parameters. The output was used to determine the Bayes factor of the number of free rate parameters. Bayes factors can be calculated as the ratio of posterior to prior odds (Pagel & Meade, 2006). The posterior odds for a set of models with *r* different rate parameters are the ratio of how often the Markov chain visited models with *r* different rates over the number of visits to all models with a number of rates other than *r*. The prior odds for a set of models with *r* different transition rates equals *N_r_*/(*N* – *N_r_*), where *N_r_* denotes the total number of models with *r* different rates and *N* denotes the total number of models. There are five trait states in the multi-state model and therefore 20 different rates between states. Since *BayesTraits* also includes the possibility for each rate to be equal to zero, *N_r_* equals

$$N_{r}=\sum_{i=r}^{20} \left( \begin{matrix} 20 \\ 20-r \end{matrix} \right)S_{2}(i, r)$$

where $S_{2}(i, r)$ denotes the Stirling number of the second kind and gives the number of combinations of grouping *i* objects into *r* classes. The derivation for *N*, the total number of models, is provided by Pagel and Mead (2006) and equals in the case of a five-state trait

$$N=B_{20}+\sum_{i=1}^{19} \left( \begin{matrix} 20 \\ 20-i \end{matrix} \right)B_{i}$$

where *B_i_* indicates the *i*^th^ Bell number.
